# An Account of the Cultivation and Preparation of Aloes, in the Island of Barbadoes

**Published:** 1787

**Authors:** L. Millington, Joseph Banks

**Affiliations:** Bart. P. R. S.


VIII. *An Account of the Cultivation and Preparation of Aloes, in the Iſland of Barbadoes; in a Letter from* L. Millington, *Eſq. to* Joſhua Steele, *Eſq. Communicated to Dr.* Simmons *by Sir* Joſeph Banks, *Bart. P. R. S.*

THE lands in the vicinity of the fea, that is, from two to three miles, which are rather fubject to drought than otherwife, and are fo ftony and fhallow as not to admit of the planting of fugar canes, with any profpect of fuccefs, are generally found to anfwer beft for the aloe plant. The ftones, at leaft the larger ones, are firft picked up, and either packed in heaps, upon the moft fhallow, barren fpots, or laid round the field as a dry wall. The land is then lightly ploughed, and very carefully cleared of all noxious weeds, lined at one foot diftance from row to row, and the young plants fet, like cabbages, at about five or fix inches from each other.

This

This regular mode of lining, and fetting the plants, is practifed only by the moft exact planters, in order to facilitate the weeding of them, by hand, very frequently, becaufe if they are not kept perfectly clean, and free from weeds, the produce will be but very fmall.

They will bear being planted in any feafon of the year, even in the drieft, as they will live on the furface of the earth, for many weeks, without a drop of rain. The moft general time, however, of planting them is from April to June. In the March following the labourers carry a parcel of tubs and jars into the field, and each takes a flip or breadth of it, and begins by laying hold of a bunch of the blades, as much as he can conveniently grafp with one hand, while with the other he cuts it juft above the furface of the earth, as quickly as poffible (that the juice may not be wafted), and then places the blades in the tub, bunch by bunch, or handful by handful.

When the firft tub is thus packed quite full, a fecond is begun, (each labourer having two) and, by the time the fecond is filled, all the juice is generally drained out of the blades in the firft tub. The blades are then lightly taken out, out, and thrown over the land, by way of manure; and the juice is poured out into a jar. The tub is then filled again with blades, and fo alternately till the labourer has procured his jar full, or about four gallons and an half of juice, which is often done in fix or feven hours, and he has then the remainder of the day to himfelf, it being his employer's intereft to get each day's operation as quickly done as poffible.

I fhould obferve, that although aloes are often cut in nine, ten, or twelve months after being planted, they are not in perfection till the fecond and third year; and that they will be productive for a length of time, fay ten or twelve years, or even for a much longer time, if good dung, or manure of any kind, is ftrewed over the field once in three or four years, or oftener if convenient.

The aloe juice will keep for feveral weeks without injury. It is, therefore, not boiled till a fufficient quantity is procured to make it an object for the boiling houfe. In the large way, three boilers, either of iron or of copper, are placed to one fire, though fome have but two, and the fmall planters only one. The boilers are filled with the juice, and as it ripens, or becomes more inſpiſſated, by a conſtant but regular fire, it is ladled forward from boiler to boiler, and freſh juice is added to that fartheſt from the fire, till the juice in that neareſt to the fire (by much the ſmalleſt of the three, and commonly called by the name of *tatch*, as in the manufactory of ſugar) becomes of a proper conſiſtency to be ſkipped or ladled out into gourds or other ſmall veſſels uſed for its final reception. The proper time to ſkip or ladle it out of the tatch is when it is arrived at what is termed a reſin height, or when it cuts freely, or in thin flakes, from the edges of a ſmall wooden ſlice, that is dipped from time to time into the tatch for that purpoſe. A little lime water is uſed by ſome aloe boilers, during the proceſs, when the ebullition is too great.

As to the ſun-dried aloes, (which is moſt approved for medicinal purpoſes) very little is made in Barbadoes. The proceſs is, however, very ſimple, though extremely tedious. The raw juice is either put into bladders, left quite open at top, and ſuſpended in the ſun, or in broad ſhallow trays of wood, pewter, or tin, expoſed alſo to the ſun, every dry day, until all the fluid parts are exhaled, and a perfect resin formed, which is then packed up for use or for exportation.

*Barbadoes-River Plantation,*
May 20th, 1787.

